# The expanding world of extracellular traps: not only neutrophils but much more

**DOI:** 10.3389/fimmu.2012.00420

**Published:** 2013-01-11

**Authors:** Oliver Goldmann, Eva Medina

**Affiliations:** Infection Immunology Research Group, Helmholtz Centre for Infection ResearchBraunschweig, Germany

**Keywords:** extracellular traps, neutrophils, mast cells, eosinophils, macrophages/monocytes, etosis

## Abstract

The release of extracellular traps (ETs) is a recently described mechanism of innate immune response to infection. Although ETs have been intensely investigated in the context of neutrophil antimicrobial effector mechanisms, other immune cells such as mast cells, eosinophils, and macrophages can also release these structures. The different ETs have several features in common, regardless of the type of cells from which they originated, including a DNA backbone with embedded antimicrobial peptides, proteases, and histones. However, they also exhibit remarkable individual differences such as the type of sub-cellular compartments from where the DNA backbone originates (e.g., nucleus or mitochondria), the proportion of responding cells within the pool, and/or the molecular mechanism/s underlying the ETs formation. This review summarizes the knowledge accumulated in recent years regarding the complex and expanding world of ETs and their role in immune function with particular emphasis on the role of other immune cells rather than on neutrophils exclusively.

## INTRODUCTION

Extracellular traps (ETs) were first described in 2004 in a ground breaking publication by Brinkmann and colleagues who observed the released of web-like structures by neutrophils after stimulation with phorbol myristate acetate (PMA), lipopolysaccharides (LPS), interleukin 8 (IL-8), platelet-mediated neutrophil activation (Clark et al., 2007) and after exposure to Gram-positive or Gram-negative bacteria (Brinkmann et al., 2004). The composition of these structures has been intensively investigated during recent years. Besides the backbone formed by DNA and histones, ETs also comprise a number of molecules which impart an antimicrobial effect including elastase, cathepsin G, proteinases or defensins, bacterial permeability increasing protein (BPI), or myeloperoxidase (MPO; Brinkmann et al., 2004; Papayannopoulos et al., 2010).

In recent years it has become increasingly evident that ETs are not formed exclusively by neutrophils but also by other cell types including, mast cells (von Kockritz-Blickwede et al., 2008), eosinophils (Yousefi et al., 2008), chicken heterophils (Chuammitri et al., 2009), and macrophages/monocytes (Chow et al., 2010). The molecules, microorganisms, and microbial products that are able to induce ETs formation by various cell types are summarized in **Table 1**.

**Table 1:** Differences between netosis, apoptosis, and necrosis.

Necrosis	Apoptosis	Netosis
Membrane and organelle disintegration	Membrane blebbing	Vacuolization
Phosphatidylserine exposure during early steps of necrosis	Phosphatidylserine exposure	No exposure to Phosphatidylserine
Cellular swelling and bursting	Nuclear chromatin condensation without disintegration of the nuclear membrane	Nuclear chromatin decondensation with disintegration of the nuclear membrane
Cell damage releasing the intracellular contents	Programmed cell death	Programmed cell death

Apart from humans and mice, ETs have also been found to be released by cells from a variety of other animals including ox, horses, fish, cats, and even by invertebrates. In fact, extracellular nucleic acid released by oenocytoid cells has been reported to be an important defense mechanism toward pathogenic microorganisms in insects (Altincicek et al., 2008). ETs are also apparent in plants where they have been demonstrated to play an important role in defense against fungal infections of the root tip (Wen et al., 2009; Hawes et al., 2011). The common feature of ETs released by the different cell types is a backbone composed of DNA decorated with antimicrobial molecules that is capable of snaring and killing a wide spectrum of microbes (Brinkmann et al., 2004; Fuchs et al., 2007; Urban and Zychlinsky, 2007; von Kockritz-Blickwede and Nizet, 2009). Nevertheless, it should be mentioned that ETs arising from different cell types also exhibit unique features, distinct from those originally described for neutrophils.

Much of the research on ETs has been conducted on neutrophils, most probably because these cells were the first to be associated with the production of such extracellular structures. This is also the reason why the mechanism of cell death leading to the formation of ETs was first termed Netosis (Fuchs et al., 2007) and then later generalized to Etosis. The differences between Etosis and the other forms of cell death such as necrosis or apoptosis are summarized in **Table 2**. The intracellular signaling events reported to be involved in the induction of etosis includes the activation of NADPH oxidase with the concomitant formation of reactive oxygen radicals (ROS; Papayannopoulos et al., 2010; Guimaraes-Costa et al., 2012). There are also reports demonstrating that, in addition to chromosomal DNA, mitochondrial DNA could also be used by eosinophils (Yousefi et al., 2008) and neutrophils (Yousefi et al., 2009) to form ETs without induction of cell death. However, the mechanism/s behind this unusual mode of ET formation remains a mystery. Although the primary function of ETs has been attributed to their antimicrobial effect, the overall role of ETs in host defense against pathogens remains a topic of debate.

**Table 2:** Cell types shown to release ETs and triggering stimuli.

Cell type	Activating agent	Reference
Neutrophils	IL-8	Ramos-Kichik et al. (2009)
Neutrophils, Mast cells	Lipopolysaccharide (LPS)	Brinkmann et al. (2004), von Kockritz-Blickwede et al. (2008), Ramos-Kichik et al. (2009)
Neutrophils, Mast cells	Phorbol-12-myristate-13-acetate (PMA)	Brinkmann et al. (2004), von Kockritz-Blickwede et al. (2008)
Neutrophils	Platelet via TLR4	Clark et al. (2007)
Neutrophils, Eosinophils	Interferon (IFN)γ + C5a	Yousefi et al. (2008)
Eosinophils	Interferon (IFN)α + C5a	Yousefi et al. (2008)
Eosinophils	Interferon (IFN) + eotaxin	Yousefi et al. (2008)
Neutrophils	GM-CSF + C5a	Martinelli et al. (2004), Yousefi et al. (2009)
Neutrophils	GM-CSF + LPS	Martinelli et al. (2004), Yousefi et al. (2009)
Neutrophils	Lipophosphoglycan	Guimaraes-Costa et al. (2009)
Neutrophils, Mast cells	M1-protein-fibrinogen complex	Lauth et al. (2009), Oehmcke et al. (2009)
Neutrophils, Mast cells, Eosinophils	Hydrogen peroxide	Brinkmann et al. (2004), von Kockritz-Blickwede et al. (2008), Oehmcke et al. (2009)
Neutrophils	Calcium	Wang et al. (2009)
Neutrophils, Mast cells	Glucose oxidase	Fuchs et al. (2007), von Kockritz-Blickwede et al. (2008)
Mast cells	IL-23 and IL-1β	Lin et al. (2011)
Neutrophils, Monocytes/Macrophages	Statins	Chow et al. (2010)
Neutrophils	Tumor necrosis factor (TNF)α	Wang et al. (2009)
Neutrophils	Panton-Valentin leukocidin	Pilsczek et al. (2010)
Neutrophils	Platelet activating factor	Hakkim et al. (2011)

## THE MOLECULAR BASIS OF EXTRACELLULAR TRAPS FORMATION

While significant progress has been made in unraveling the cellular processes that are taking place during the formation of ETs, many aspects still remain unresolved. ET formation generally begins in stimulated cells with the loss of the tight organization of the nuclei followed by chromatin decondensation. The characteristic shape of the nuclei disappears and a gap between the inner and outer membrane of the nucleus emerges. Formation of vesicles in the nuclear membrane follows leading to widespread membrane disruption. At the same time, disruption of the granular membranes takes place in the cell cytoplasm facilitating the mixing of granular content with the chromatin leaking into the cytoplasm through the disrupted cellular membrane. Finally, eruption of the cell membrane follows and DNA mixed with the granular content is released into the extracellular milieu (Fuchs et al., 2007). This characteristic form of cell death, termed Netosis by Steinberg and Grinstein (2007), was described earlier by Takei et al. (1996) although without showing an association with the release of ETs. Netosis seems to be a process entirely independent of caspases and certain kinases such as RIP-1 and is not affected by the caspase inhibitor zVAD-fmk (Urban et al., 2009; Remijsen et al., 2011). Netosis is not associated with DNA fragmentation or phosphatidylserine (PS) exposure on the outer leaflet of the cellular membrane, which are distinctive aspects of apoptosis. The lack of PS impedes the clearance of cells undergoing netosis by phagocytic cells such as macrophages. An additional feature that distinguishes netosis from apoptosis and necrosis is the fact that both the nuclear as well as the granular membranes undergo fragmentation.

**FIGURE 1:**
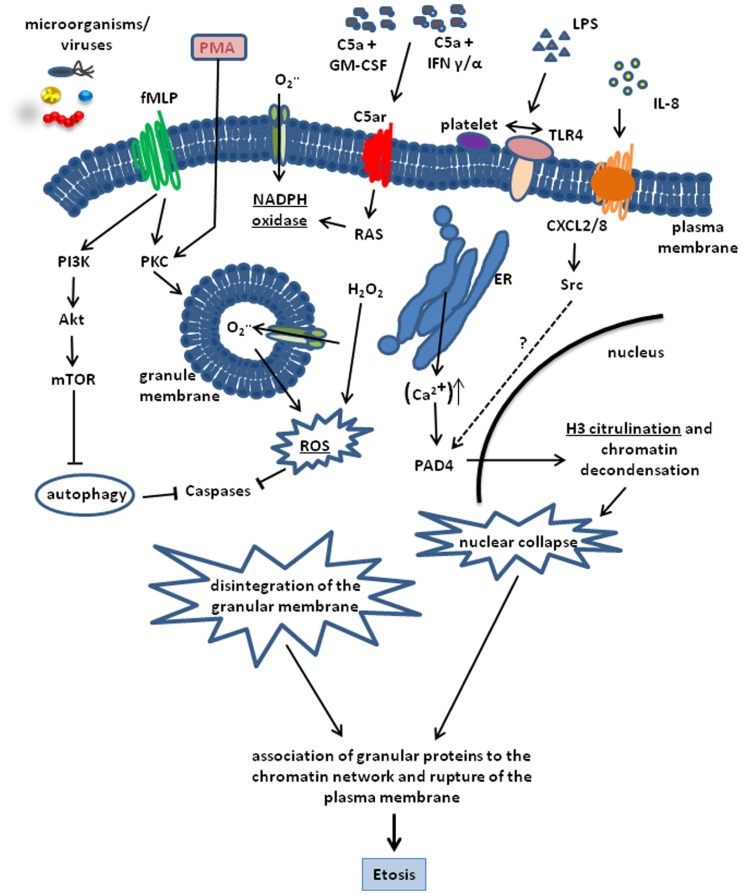
**Schematic representation of the cellular processes involved in the formation of ETs**. The process can be triggered by a number of stimuli including, PMA, LPS, C5a + GM-CSF, IFN α/γ. LPS, bacteria, and viruses. IL-8 is also able to trigger ET release by interacting with the CXCL2/8 receptor and inducing H3 citrullination through PAD4 activation via Src kinases. Most pathways converge in the activation of the key enzyme NADPH oxidase. This enzyme is highly activated by PMA and formylated peptides. Induction of the fMLP receptor leads to a massive activation of protein kinase C (PKC) and NADPH oxidase activity. On the other hand, fMLP blocks autophagy via PI3K, Akt and mTOR activation, which is able to prevent Etosis. NADPH oxidase activity results in ROS production and H3 citrullination leading to chromatin decondensation and nuclear collapse. Disintegration of the nuclear membrane and adsorption of antimicrobial granular proteins onto the decondensed chromatin network is the final step of Etosis that precedes the release of ETs into the surrounding milieu after rupture of the plasma membrane.

A critical factor involved in etosis and ET formation is the production of ROS. In neutrophils, ROS produced by NADPH oxidases has been reported to inactivate caspase function thereby leading to the blockage of the apoptotic cell death pathway (Fadeel et al., 1998; Hampton et al., 2002). The importance of NADPH oxidase for ET release was demonstrated by the reduced capacity of neutrophils to form ETs after pharmacological inhibition of this enzyme (Metzler et al., 2011). Furthermore, neutrophils from patients suffering from chronic granulomatous diseases, which are defective in NADPH oxidase function, are unable to form ETs (Fuchs et al., 2007; Bianchi et al., 2009). Etosis is nevertheless a multifactorial process and NADPH oxidase activity is necessary but alone is insufficient to trigger this process. Thus, increased intracellular Ca^2^^+^ levels after treatment with Thapsigargin has also been shown to induce ET formation in neutrophils (Gupta et al., 2010). The increased Ca^2^^+^ level induces a Ca^2^^+^-dependent PAD4 activity leading to histone citrullination, which constitutes a downstream signaling processes in the formation of ETs (Neeli et al., 2008; Wang et al., 2009). Indeed, PAD4-dependent citrullination of histone H3 is a key molecular event in the formation of ETs (Neeli et al., 2008; Wang et al., 2009; **Figure 1**).

Even though the development of ROS and the activity of NADPH oxidase have been claimed as being essential in the formation of ETs, it has also been reported that microorganisms such as *Staphylococcus aureus* (Pilsczek et al., 2010) or *Leishmania donovani* (Gabriel et al., 2010) are able to induce ET release through a molecular process that is independent of ROS. This adds a further level of complexity to the molecular puzzle of this cellular process.

## EXTRACELLULAR TRAPS FORMATION OUTSIDE THE NEUTROPHIL WORLD

As mentioned before, other immune cells including mast cells, eosinophils, and macrophages are also capable of releasing ETs. Although the molecular principles underlying the formation of ETs by mast cells (von Kockritz-Blickwede et al., 2008), eosinophils (Yousefi et al., 2008), and monocytes/macrophages (Chow et al., 2010) share some similarities with those observed for neutrophils, there are some notable disparities. The most remarkable mechanism of ET formation has been described in eosinophils. In these cells, ETs are formed by both nuclear and mitochondrial DNA in a ROS-dependent manner. The presence of several mitochondrial genes including Co1 (cytochrome oxidase subunit 1), ND1 (NADH dehydrogenase subunit 1), or Cyb (cytochrome *b*) in the nucleic acid material released by eosinophils provides clear evidence of its mitochondrial origin (Yousefi et al., 2008). DNA is rapidly expelled by the eosinophils in response to stimulation with LPS, eotaxin, complement factor 5a (C5a) or infection with Gram-negative bacteria after priming with interleukin 5 (IL-5) or IFN-γ (**Figure 1**), which, in this case, was shown to be essential for the explosive release of mtDNA by eosinophils. The time frame reported for the release of eosinophil ETs is seconds and is thus much shorter than the classical ET formation by neutrophils. An additional and interesting characteristic of ETs formed by eosinophils is the lack of cytosolic proteins, although eosinophil granule proteins were shown to be released concurrently with mtDNA (Yousefi et al., 2012). An additional and important feature that differentiates the eosinophil from the classical neutrophil release of ETs is that it is not dependent upon the cell death of the eosinophils. Interestingly, a similar mechanism of ET release that is non-associated with cell death has also been recently described for neutrophils (Pilsczek et al., 2010). This challenges the generalized opinion that ETs are released by dying cells. Nevertheless, it should also be noted that in both cases where ET formation was non-associated with cell death, the cells needed to be primed first before stimulated to form ETs. In the case of neutrophils, cells were initially activated by granulocyte/macrophage stimulating factor (GM-CSF) followed by short-term toll-like receptor 4 (TLR4) or C5a stimulation (**Figure 1**). In these experimental conditions, viable neutrophils were able to release ETs that contained mitochondrial but no nuclear DNA.

DNA-releasing eosinophils have been primarily reported in the context of inflammatory diseases of the intestine (Yousefi et al., 2008) and skin (Simon et al., 2011). They seem to be less prominent, however, in the setting of infectious diseases despite the fact that these structures are also capable of snaring and killing bacteria (Yousefi et al., 2008). Furthermore, while induction of mtDNA associated with eosinophil granules has been reported to contribute to the increased survival of mice (up to 14 days) undergoing cecal ligation puncture (CLP; Yousefi et al., 2008), it is still not clear to what extent eosinophil ET formation contributes to host defense. In this regard, though evidence has been provided that hypereosinophilic transgenic animals are less susceptible to septicemia induced by CLP, the major role of eosinophils has been attributed to host defense against helminths (Blanchard and Rothenberg, 2009; Linch et al., 2009). These granulocytic cells are able to infiltrate the gastrointestinal tract and have been associated with a variety of inflammatory conditions like inflammatory bowl disease (IBD) or eosinophil-associated gastrointestinal disorders (EGIDs; DeBrosse and Rothenberg, 2008; Wedemeyer and Vosskuhl, 2008).

**FIGURE 2:**
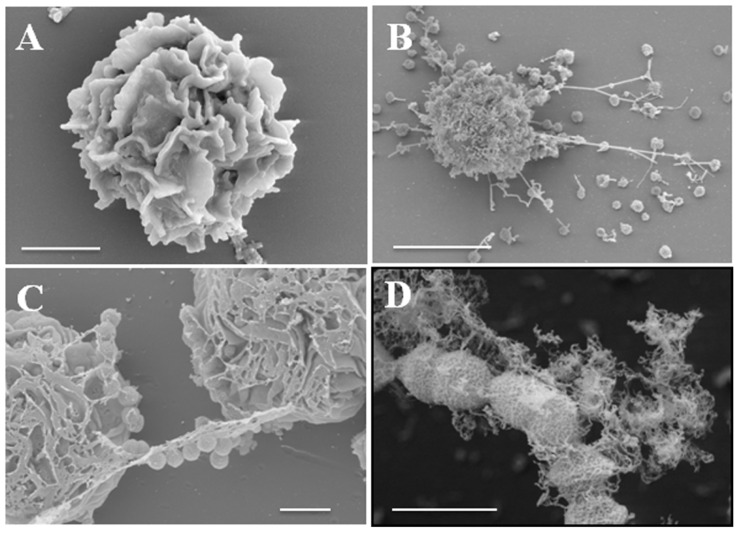
**Release of ETs by mast cells after encounter with *S. pyogenes*.** **(A)** Field-emission scanning electron microscope (FESEM) images of a resting mast cell (bar, 2 μm). **(B,C)** Mast cells in the process of releasing ETs in response to *S. pyogenes* (**B**, bar, 5 μm and **C**, bar, 2 μm). **(D)** *S. pyogenes* captured in ETs (bar, 1 μm). Provided by M. Rohde, Department of Microbial Pathogenesis, Helmholtz Center for infection Research, Braunschweig, Germany.

Besides eosinophils, mast cells, which also originate from bone marrow and contain different types of granules enclosing very potent biological effectors molecules, are also capable of releasing ETs following stimulation (**Figure 2**). Mast cells are located in close proximity to the host environment where they are most likely to encounter incoming pathogens. Although mast cells are largely known for their detrimental role in the context of allergic diseases, there is a growing body of evidence that suggests that they are also important contributors to host defense against pathogens (Galli and Wershil, 1996; Bischoff, 2007). Thus, mast cells are not only important for modulating the function of other immune cells (e.g., neutrophils) during infection but they also impart direct antimicrobial effects (Feger et al., 2002). Due to the limited phagocytic activity of mast cells, their antimicrobial activity is largely mediated by extracellular mechanisms including degranulation and the concomitant release of highly potent antimicrobial peptides such as cathelicidins (CRAMP or LL-37), defensins (β-defensins) or proteases (tryptase, chymase). Mast cell degranulation occurs after exposure to pathogens and has been shown to be very efficient in inhibiting the growth of bacteria such as *S. aureus* (Abel et al., 2011). In addition, mast cells are also able to release ETs in a ROS-dependent manner. Mast cell ETs are composed of DNA and histones, which are the general components of most ETs, as well as mast cell-specific granule proteins like tryptase and CRAMP/LL-37 (von Kockritz-Blickwede et al., 2008). In contrast to neutrophils where NETs can be dismantled after treatment with only DNase, the complete disassembling of mast cell ETs requires treatment with DNase as well as the addition of enzymes degrading tryptase (e.g., MPO; von Kockritz-Blickwede et al., 2008). Another interesting feature is the recently reported involvement of the transcriptional hypoxia-inducible factor 1α (HIF-1α) in the modulation of ET release by human and murine mast cells (Branitzki-Heinemann et al., 2012). HIF is a well-known factor for its role in the regulation of the inflammatory and innate immune function of neutrophils and macrophages (Cramer et al., 2003; Peyssonnaux et al., 2005).

Most recently, monocytes/macrophages have also been reported to be capable of releasing ETs (Chow et al., 2010; Aulik et al., 2011). Macrophage ET production has been shown to be boosted by statins, which are inhibitors of the rate-limiting enzyme within the cholesterol biosynthesis 3-hydroxy 3-methyglutaryl coenzyme A (HMG-CoA) reductase. In addition, increased production of ETs release by macrophages has been observed after inhibition of HMG-CoA reductase using siRNA or after treatment of macrophages with the downstream HMG-CoA reductase product mevalonate (Chow et al., 2010). Statins are also capable of inhibiting the release of ETs by neutrophils. The molecular mechanism mediating the effect of statins on phagocytes seems to be linked to the inhibition of the sterol pathway within the cell (Chow et al., 2010). Interestingly, bacterial components such as hemolysins of *Escherichia coli* or leukotoxin of *Mannheimia haemolytica* have been shown to induce the release of ETs by bovine macrophages (Aulik et al., 2011, 2012). However, the extent to which the molecular processes leading to the formation of ETs by monocytes/macrophages is comparable to the mechanisms already described for neutrophils, eosinophils, and mast cells, remains to be elucidated. Although little information is available regarding the molecular basis of ET release by macrophages, it seems that general mechanisms such as NADPH oxidase dependency and oxidative stress are involved (Chow et al., 2010).

## THE ANTIMICROBIAL EFFECT OF EXTRACELLULAR TRAPS

Extracellular traps release is thought to be mainly an antimicrobial strategy used by host cells to control and eliminate pathogens (Brinkmann et al., 2004; von Kockritz-Blickwede et al., 2008; Linch et al., 2009; Saitoh et al., 2012). Thus, a number of bacteria, fungi, and parasites have been reported in the literature to be entrapped and killed by ETs (summarized in **Table 3**). Saitoh et al. (2012) provided the first report regarding the involvement of ETs in antiviral immunity. Their study showed that neutrophils are able to produce ETs in response to human immunodeficiency virus-1 (HIV-1) and, more interestingly, that these virus particles can be entrapped and neutralized by the ETs. By using blocking antibodies to MPO and α-defensin, it was possible to demonstrate that the viral neutralization was dependent on the presence of MPO and α-defensin in the NET structure. The production of NETs by neutrophils in this case was associated with TLR-7 and TLR-8 signaling as well as with ROS production (Saitoh et al., 2012). In addition, the investigators also showed that the anti-inflammatory cytokine IL-10 could reduce the release of extracellular DNA by neutrophils into the surrounding milieu. It is important to note, however, that these studies were carried out *in vitro* and, although this is an exciting new aspect of ET function in host immunity, it still remains to be demonstrated in the *in vivo* setting. Whether ETs produced by immune cells other than neutrophils are also capable of trapping and inactivating virus particles may be deserving of future investigation. The finding that different pathogens are able to induce ETs in different innate immune cells argues for a general role of ETs in the innate immune response to pathogenic microorganisms and is supported by a number of *in vivo* studies revealing ET formation in necrotizing soft tissue infections caused by *S. pyogenes* (Buchanan et al., 2006), polymicrobial sepsis after cecal ligation and puncture (Yousefi et al., 2008) and *S. pneumoniae* infections in murine models (Buchanan et al., 2006).

**Table 3:** Microorganisms able to trigger the release of ETs by specific cell types.

Microorganism	Cell type	Reference
*Aspergillus fumigatus*	Neutrophils	Bruns et al. (2010), McCormick et al. (2010)
*Candida albicans*	Neutrophils	Urban et al. (2006)
*Cryptococcus gattii *	Neutrophils	Springer et al. (2010)
*Cryptococcus neoformans*	Neutrophils	Urban et al. (2009)
*Eimeria bovis*	Neutrophils	Behrendt et al. (2010)
*Enterococcus faecalis*	Neutrophils	Lippolis et al. (2006)
*Escherichia coli*	Neutrophils, monocytes	Lippolis et al. (2006), Grinberg et al. (2008), Webster et al. (2010)
*Haemophilus influenzae*	Neutrophils	Hong et al. (2009), Hakkim et al. (2011)
*Helicobacter pylori*	Neutrophils	Hakkim et al. (2011)
*Human Immunodeficiency Virus-1 (HIV-1)*	Neutrophils	Saitoh et al. (2012)
*Klebsiella penumoniae*	Neutrophils	Papayannopoulos et al. (2010)
*Leishmania amazonensis*	Neutrophils	Guimaraes-Costa et al. (2009)
*Listeria monocytogenes*	Neutrophils	Ermert et al. (2009)
*Mycobacterium canettii*	Neutrophils	Ramos-Kichik et al. (2009)
*Mycobacterium tuberculosis*	Neutrophils	Ramos-Kichik et al. (2009)
*Pseudomonas aeruginosa*	Mast cells	von Kockritz-Blickwede et al. (2008)
*Serratia marcescens*	Neutrophils	Lippolis et al. (2006)
*Shigella flexneri*	Neutrophils	Brinkmann et al. (2004)
*Staphylococcus aureus*	Neutrophils, Mast cells	Brinkmann et al. (2004), von Kockritz-Blickwede et al. (2008)
*Streptococcus dysgalactiae*	Neutrophils	Lippolis et al. (2006)
*Streptococcus pneumoniae*	Neutrophils, Mast cells	Beiter et al. (2006), Crotty Alexander et al. (2010)
*Streptococcus pyogenes*	Neutrophils, Mast cells	Buchanan et al. (2006), von Kockritz-Blickwede et al. (2008)

The molecular mechanism/s responsible for the entrapment and killing of microorganisms within ETs is a matter of debate, though several hypotheses have been proposed. One such hypothesis is that entrapment is facilitated by the occurrence of electrostatic interactions arising from the cationically charged ET structure and the anionically charged bacterial surfaces (Brinkmann and Zychlinsky, 2007). The subsequent killing of the pathogen is postulated to arise from the ability of the ETs to increase the local concentration of certain antimicrobial peptides and therefore intensifying the contact between microorganisms and the antimicrobial agents (von Kockritz-Blickwede et al., 2008). Potential candidates being discussed to have antimicrobial properties within ETs are the histones. Several types of histones and histone-related peptides isolated from various organisms and cell types exhibit a broad spectrum of antimicrobial activities (Kawasaki and Iwamuro, 2008). In particular, the histone H2B displays antimicrobial properties against Gram-positive and Gram-negative bacteria and fungi (Li et al., 2007). An overview of the antimicrobial activities of histones is displayed in **Table 4**. In addition to histones, there are other cell specific components located within the ETs that may have antimicrobial effect. The two most important antimicrobial peptide families in mammals are the defensins (Lehrer and Ganz, 2002b) and a group of cationic molecules, classified as cathelicidins (Lehrer and Ganz, 2002a; Zaiou and Gallo, 2002). Cathelicidins belong to a family of antimicrobial peptides found in the lysosomes of several immune cells including neutrophils, mast cells, and macrophages (Nizet et al., 2001; Zanetti, 2004). The presence of these antimicrobial peptides has been demonstrated in ETs released by neutrophils as well as by mast cells (Brinkmann et al., 2004; von Kockritz-Blickwede et al., 2008). However, the question remains whether antimicrobial peptides bound to the DNA backbone of the ETs still retain their antimicrobial capacity.

**Table 4:** Short overview of histones and their antimicrobial properties.

Histone	Origin	Antimicrobial spectrum	Reference
Histone H1	macrophages, epithelial cells, liver, intestine, skin	*S. aureus*, *L. moncytogenes*, *S. typhimurium*, *E. coli*, *C. neoformans*	Hiemstra et al. (1993), Rose et al. (1998)
Histone H2A	Placenta, skin, liver	*E. coli*, *S. aureus*, *B. subtilis*, *S. flexneri*, *S. typhimurium*, *S. pneumonia*, *C. albicans*	Kim et al. (2002), Cho et al. (2002), Fernandes et al. (2002), Li et al. (2007)
Histone H2B	Placenta, skin, liver	*S. aureus*, *L. moncytogenes*, *S. typhimurium*, *B. subtilis*	Li et al. (2007)

Although the antimicrobial effect of ETs has been extensively demonstrated in many experimental settings, the extent to which these structures contribute to pathogen killing during productive infection remains a subject of debate. Furthermore, in certain circumstances, the production of ETs can be detrimental for the host. For example, the release of high quantities of DNA and histones can induce autoimmune reactions that may be involved in the development of autoimmune diseases like lupus erythematosus or rheumatoid arthritis (Mohan et al., 1993; Zhong et al., 2007). Preeclampsia, a severe disorder of late pregnancy characterized by an increasing level of cell free DNA in the maternal plasma, is another pathological disorder in which ET formation may also be involved (Clark et al., 1998). In this disorder, a massive release of DNA probably in response to high levels of inducing factors (e.g., IL-8 or microdebris of the placenta) has been observed (Gupta et al., 2005, 2007). Similarly, the release of ETs by platelet-activated neutrophils under blood flow conditions can result in reduced blood perfusion of the tissue and ischemia (Clark et al., 2007). The beneficial or detrimental effect of ETs can be determined by the extent of the response. Moderate release of ETs during infection can contribute to pathogen killing and control of the infection, thus conferring a beneficial effect. Conversely, massive release of ETs during pathological conditions can induce autoimmunity as well as organ damage and is thus highly deleterious for the host.

## PATHOGEN EVASION OF EXTRACELLULAR TRAPS

Successful pathogens have evolved intricate countermeasures to subvert the mechanisms of host defense. Shortly after ETs were discovered, a number of studies reported the ability of certain pathogens to circumvent the antimicrobial activity of these structures. One of the main strategies used by pathogenic bacteria to escape the ETs is through the production of DNases that cleave DNA and therefore dismantle their DNA backbone. This mechanism has been described for *S. pyogenes*, which produces a very potent bacteriophage-encoded DNase designated Sda1. Strains of *S. pyogenes* producing Sda1 are more resistance to ET-dependent killing than strains lacking the *Sda1* gene (Sumby et al., 2005; Buchanan et al., 2006). A similar strategy has been reported for *S. pneumoniae* (Buchanan et al., 2006) and *S. aureus* (Udou and Ichikawa, 1979; Berends et al., 2010). Changes in the composition of the bacterial cell wall can also help to avoid the antimicrobial activity of ETs. Thus, *S. pneumoniae* mutant strains lacking positively charged D-alanyl residues on their lipoteichoic acid (LTA) have been shown to be more susceptible to ET killing than the corresponding wild-type strain (Wartha et al., 2007a,b). D-alanylation of LTA by bacterial species harboring a homolog of the *dlt* operon like *S. pyogenes* (Kristian et al., 2005) or *S. aureus* (Kraus et al., 2008) are known to be much more resistant against the antimicrobial activity of cathelicidins. An indirect strategy of microbes to avoid the antimicrobial effect of ETs is to reduce the recruitment of immune cells involved in the production of ETs. This is achieved by the blocking or cleaving of chemotactic mediators involved in the recruitment of immune cells to the site/s of infection. An example of this is provided by the IL-8 degrading protein SpyCEP of *S. pyogenes* (Gupta et al., 2005).

## CONCLUDING REMARKS

Despite the large number of studies that have been conducted on ETs, they still remain enigmatic structures, and many aspects regarding their nature and significance is deserving of further investigation. The specific mechanism/s responsible for pathogen entrapment by ETs is still unsolved. Although some light has been shed regarding the killing mechanisms employed by ETs, the actual process is still largely unknown and requires detailed exploration. In particular, the role of antimicrobial agents like cathelicidins or histones is still under discussion. The extent to which the binding of these molecules to DNA may alter their biological functionality is also unknown. Another question that remains open is related to why only a small proportion of cells within the total population are primed to release ETs. This argues against a primary role of ETs in the functional biology of these cells. Perhaps, a major function of ETs is to contain the pathogen at the site of infection, thereby limiting its spread and dissemination. Indeed, this is a feature also ascribed in the late 1980s to fibrin networks where it was also demonstrated that they were able to interfere with the phagocytic function of neutrophils by blocking effective phagocytosis (Bruns et al., 2010). An additional problem in investigations of the release of ETs is the high variability of experimental settings employed by different laboratories. For example, the concentration of PMA used in different studies ranges from 20 to 200 μM. Furthermore, *in vitro* growth conditions such as nutrient and serum supplementation as well as the time frame for induction are heterogeneous in the literature. This variability may lead to incorrect assumptions and serious misinterpretations.

Future research should be directed to addressing the limitations of these investigations and detailing the signaling pathways leading to etosis. More importantly, further insights into the mechanism/s underlying the regulation of etosis are required. This is of particular importance given that the process of cell death releases many biologically active components that may be both beneficial but also detrimental to the host.

## Conflict of Interest Statement

The authors declare that the research was conducted in the absence of any commercial or financial relationships that could be construed as a potential conflict of interest.
